# Three-Dimensional Micromechanical Modeling of Martensite Particle Size Effects on the Deformation Behavior of Dual-Phase Steels

**DOI:** 10.3390/ma17205004

**Published:** 2024-10-13

**Authors:** Onur Cavusoglu, Serkan Toros

**Affiliations:** 1 Department of Manufacturing Engineering, Faculty of Technology, Gazi University, Teknikokullar, 06560 Ankara, Türkiye; 2 Department of Mechanical Engineering, Faculty of Engineering, Nigde Omer Halisdemir University, 51240 Nigde, Türkiye; serkantoros@ohu.edu.tr; 3 Turkish Aerospace Industries Inc., 06980 Ankara, Türkiye

**Keywords:** dual-phase steel, particle size, martensite, 3D micromechanical analysis, RVE, representative volume elements

## Abstract

The objective of this study was to examine the influence of martensite particle size on the formation of stress and strain in microstructures of dual-phase steels. In order to achieve this objective, the 3D representative volume element (RVE) method was utilized. Particle size distributions were obtained from the microstructures of DP600 and DP1000 dual-phase steels as they actually exist. Virtual dual-phase steel microstructures were generated according to the above distribution and subsequent validation analyses were performed. In the subsequent phase, microstructures of varying martensite particle sizes (1 µm, 1.98 µm, 3 µm for DP600 and 1.15 µm, 2 µm, 3 µm for DP1000) were formed, and the effects of particle size on deformation behavior under tensile loads were determined. The findings indicated that an increase in martensite particle size resulted in a reduction in tensile strength, accompanied by an increase in deformation amount.

## 1. Introduction

Advanced high-strength steels (AHSS) have been developed with the objective of minimizing fuel consumption and carbon footprints by reducing vehicle weights, and are currently the preferred choice within the automotive industry [1,2,3]. Among these steels, dual-phase steels have emerged as a significant area of interest. The high strength of the structural components of vehicles is associated with enhanced formability due to the low yield strength, which contributes to passenger safety. Dual-phase steels are among the most widely used in the AHSS family [4,5,6]. The microstructure of dual-phase steels is composed of martensite and ferrite phases, together creating an intricate interplay between strength and ductility. The production of dual-phase steels is achieved through the heating of the material to the α+γ regions, followed by a rapid cooling process that enables the transfer of the austenite phase to the martensite phase. During the martensitic transformation, a diffusionless phase change occurs, whereby the face-centered cubic (FCC) structure of austenite shifts to a body-centered tetragonal (BCT) martensite structure. The aforementioned transformation gives rise to internal stresses as a consequence of the dissimilar lattice structures, which in turn result in an enhanced dislocation density and increased hardness in the martensite phase [7,8,9]. The martensite phase is rich in carbon, thereby providing the strength of steel. In contrast, the ferrite phase gives steel ductility [10]. The volume fraction and structures of the phases present in the steel content may vary depending on the heat treatment and cooling process, which in turn determines the characteristics of the steel [11]. The distribution of martensite particles within the ferrite phase, the size and morphology of the phases, and the mechanical properties of the material are all influenced by one another [12,13].

Finite element analysis tools have revolutionized the modeling of steel microstructures, providing an avenue to study mechanical properties without physical production. The microstructure of steel can be modeled with finite element analysis software developed in recent years. The mechanical properties and deformation behavior of steel can be determined according to the particle size, grain structure, and phase volume fraction, without the necessity of steel production. In accordance with the material properties, the microstructures that are generated within the computer environment can be analyzed through the use of the representative volume element (RVE) method. In the literature, Arezodar and Nikbakht conducted a deformation analysis on dual-phase steels with varying martensite volume fractions and particle sizes. It was observed that an increase in the martensite volume fraction and a decrease in particle size resulted in enhanced mechanical properties [12]. However, their work primarily focused on volume fraction and did not examine the specific role of martensite particle size across different sizes, which is a key aspect of this study. Anbarlooie et al. conducted an analysis of dual-phase microstructures with different martensite volume fractions, employing 2D and 3D representative volume elements [14]. Their research demonstrated the usefulness of the RVE method, but the specific effects of particle size on stress–strain behavior were not fully explored. Eghtesad and Knezevic [15] employed the 3D representative volume element method to examine the microstructures of DP590, DP980, and DP1180 dual-phase steels. Their focus was mainly on volume fraction, leaving particle size effects less explored. Hosseini-Toudeshky et al. performed an analysis of the effects of martensite volume fraction using real 2D dual-phase microstructure geometries [16]. Mangas et al. investigated the uniaxial tensile behavior of dual-phase steels with 2D representative volume elements at varying martensite volume fractions [17]. Rana and colleagues studied the cyclic plastic deformation of dual-phase steels with varying martensite volume fractions using 2D representative volume elements [18]. These 2D studies provided insights into general deformation mechanisms but lacked a detailed 3D analysis of particle size effects, which is critical for understanding microstructural behavior. The findings of these studies indicated that the level of stress experienced by the material increased in direct correlation with the martensite volume fraction. In their study, Li et al. demonstrated that an increase in carbon content results in enhanced strength, while damage occurs during deformation in the ferrite phase [19]. Matsuno et al. investigated the effect of martensite particle type by employing 3D representative volume elements and determined that the banding-type structure markedly enhanced tensile strength [20]. Basantia et al. examined the influence of representative volume element size on the prediction of tensile strength in dual-phase steel. It was demonstrated that the accuracy rate increased in conjunction with an increase in the representative volume element size [21]. While Matsuno et al. [20] and Basantia et al. [21] highlighted the importance of martensite morphology and RVE size, they left a gap in understanding the effects of varying particle sizes within a 3D context. In a study conducted by Amirmaleki et al., DP500 and DP600 dual-phase steels were analyzed using the 3D RVE method, and the results were found to be in close agreement with the experimental findings [22]. Abid et al. demonstrated that reducing the size of martensite particles has the effect of increasing tensile strength. While their study focused on a martensite particle size and lacked a detailed examination of the stress–strain relationship in 3D [23].

The preceding findings in the existing literature indicate that the microstructural deformation behavior of dual-phase steel can be effectively simulated by the representative volume element (RVE) method. The novelty of this study lies in the detailed exploration of how martensite particle size affects deformation behavior, specifically focusing on DP600 and DP1000 dual-phase steels using the 3D representative volume element (RVE) method. While previous studies have investigated various microstructural factors, they often focus on martensite volume fraction or general phase properties rather than the specific influence of particle size across a range of sizes. This work extends the understanding of particle size effects on mechanical properties, particularly in relation to stress and strain distributions.

In this study, the microstructures of DP600 and DP1000 dual-phase steel with varying martensite particle sizes were modeled using the 3D representative volume element method, and the deformation behavior was subsequently analyzed using the finite element method. The impact of martensite particle size on stress and strain distributions within microstructures and flow curves was examined through a comparative analysis.

## 2. Materials and Methods

In this study, commercial DP600 and DP1000 dual-phase steels were used as a reference point to investigate the effect of martensite particle size. The chemical compositions of the materials used in this study are shown in Table 1. To investigate the effect of martensite particle size, three different microstructures were used that included different reference particle sizes, finer particles, and coarser particles. 

**Figure 1:**
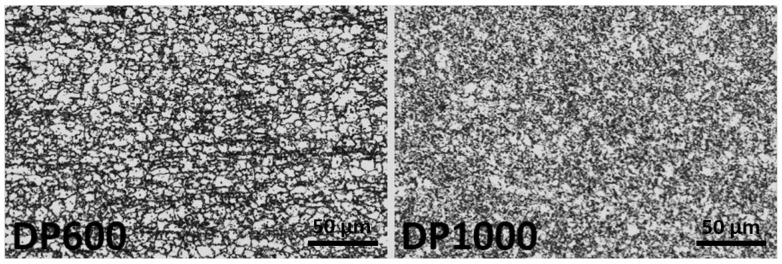
The microstructure of DP600 and DP1000 dual-phase steel.

**Figure 2:**
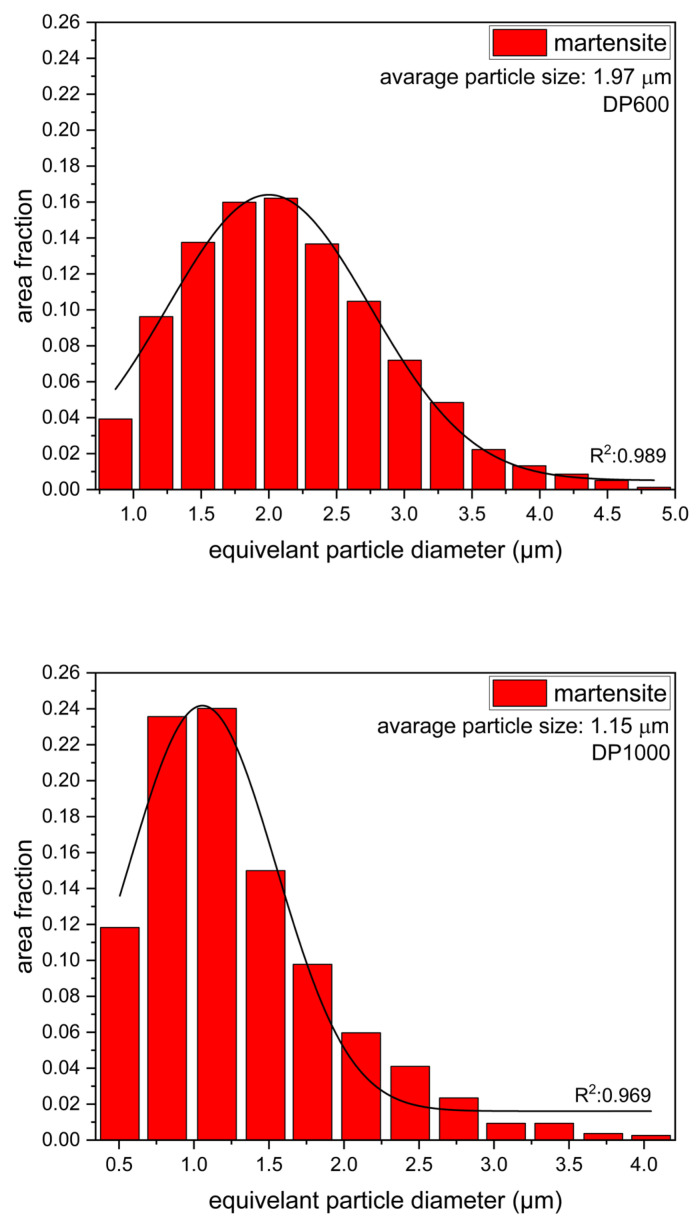
The martensite particle size distribution of DP600 and DP1000 dual-phase steel.

To determine the mechanical properties of the steels, tensile tests were performed on specimens prepared in accordance with ASTM E-8 [24]. Tensile tests were performed at room temperature with a strain rate of 0.0083 s^−^¹ using a Shimadzu (Kyoto, Japan) Autograph 100 kN tensile testing machine. The samples prepared for metallographic investigation were etched in a solution of nitric acid and ethanol (2% nital). Microstructure images at 500× magnification were obtained through the use of an optical microscope. Figure 1 illustrates the microstructure of DP600 and DP1000 dual-phase steels. The phase volume fraction and particle size distributions were calculated by means of an analysis of the multiple microstructure images, conducted using the Leica V4.9 software. The particle size distributions are illustrated in Figure 2. The phase volume fractions and martensite particle sizes of DP600 and DP1000 dual-phase steels are presented in Table 2.

## 3. Microstructure Modeling

### Virtual Microstructure Generation for 3D Representative Volume Elements

The observed deformation behavior in the microstructure can be modeled through the implementation of finite element analysis, utilizing a three-dimensional representative volume element method. Three-dimensional microstructure production was achieved through the utilization of the DREAM.3D software V.6.4.78 (Digital Representation Environment for Analyzing Microstructure in 3D) [25,26], employing the martensite particle size distributions derived from the authentic microstructure of DP600 and DP1000 dual-phase steels. In accordance with the equations for grain size distribution (GSD), outlined in Equations (1) and (2), the µ and σ values were derived from the particle size distribution. 

**Figure 3:**
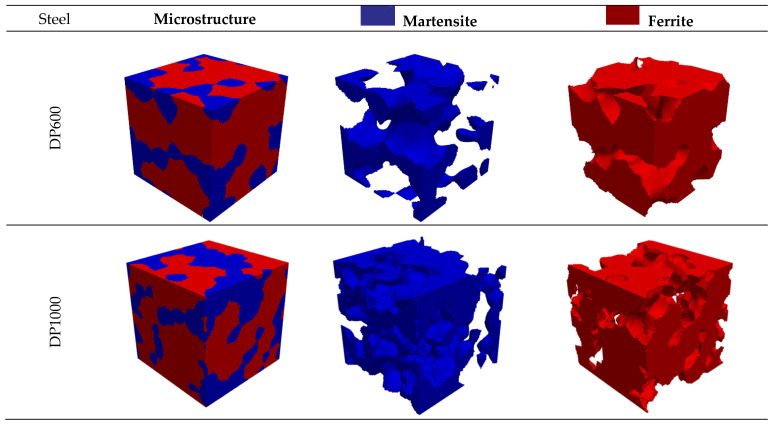
The virtual microstructure of dual-phase steel for DP600 and DP1000.

The parameter μ represents the mean of the log-normal particle size distribution, while σ denotes the mean value of the standard deviation. Furthermore, the minimum and maximum values serve as the cut-off points for σ, thereby establishing the limits for the smallest and largest particle sizes [27]. The dimensions of the microstructure were selected to be 8 × 8 × 8 µm^3^. Martensite particles were created in the range of 0–5 µm for DP600 steel and 0–4 µm for DP1000 steel. Ellipsoidal microstructures were observed to form [28]. Figure 3 depicts the virtual microstructure geometries generated in accordance with the actual microstructure data in DREAM.3D.

(1) $$GSD_{min}=X^{\mu +min*\sigma }$$

(2) $$GSD_{max}=X^{\mu +max*\sigma }$$

In dual-phase steels, such as the DP600 and DP1000 varieties, two different phases, martensite and ferrite, are present with different volume fractions [29]. Equation (3) illustrates the mathematical relationship utilized to ascertain the mechanical behavior of phases [10,30,31,32].

(3) $$\sigma =\sigma_{y}+\alpha \mathrm{MG}\sqrt{b}\sqrt{\frac{1-\exp\left(-{\mathrm{MK}}_{r}\varepsilon \right)}{K_{r}L}}$$

In this context, σ represents the flow stress, and ε represents strain. The remaining terms in the equation were derived from earlier studies [11,16]. The second term in Equation (3) accounts for both dislocation strengthening and work softening due to recovery. Here, α is a constant with a value of 0.33, M is the Taylor factor (M = 3), G is the shear modulus (G = 80 GPa), b is the Burgers vector (b = 2.5 × 10^−10^ m), and Kr is the recovery rate (for ferrite, Kr = 10^−5^/dα, and for martensite, Kr = 41), where dα (in meters) is the ferrite grain size. L represents the mean free path of the dislocations, with a value of L = 3.8 × 10^−8^ m for martensite [10,11]. The initial term in Equation (3) is equivalent to the yield stress, as defined in Equation (4), which can be expressed as follows:

(4) $$\sigma_{y}=70+37\mathrm{Mn}+83\mathrm{Si}+2918N_{\mathrm{sol}}+33\mathrm{Ni}-30\mathrm{Cr}+680P+38\mathrm{Cu}+11\mathrm{Mo}+5000C+\frac{15*1}{\sqrt{d}}$$

The first term (70 MPa) represents the stress friction value, d (mm) represents the grain size (in millimeters), and the alloy content is expressed in weight percent (wt%).

**Figure 4:**
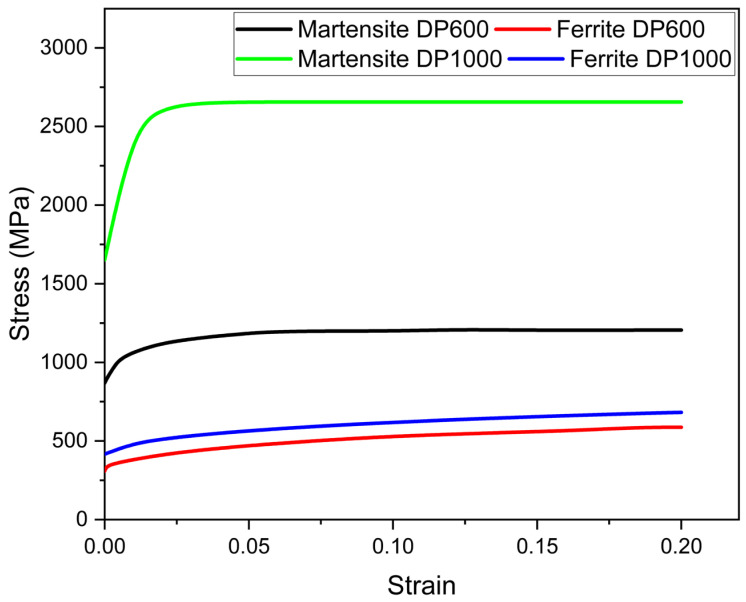
Flow curves of single phases.

The microstructure geometries were then imported into the finite element analysis software. Subsequently, the mechanical properties of each phase were incorporated. To perform a finite element analysis, the stress–strain behavior of each phase present in the microstructure must be entered into the software. The flow curves for the ferrite and martensite phases in DP600 and DP1000 steels were modeled using the mathematical relationship outlined in the constitutive description section. Figure 4 illustrates the flow curves of these phases in DP600 and DP1000 dual-phase steels. As illustrated in Figure 4, there is no notable discrepancy between the flow curves of the ferrite phase in both varieties of steel. Upon examination of the flow curves for the martensite phases, it becomes evident that the martensite phase in DP600 steel exhibits a lower stress level in comparison to that observed in DP1000 steel. The observed variation in stress levels within the martensite phase is attributed to differences in carbon content. It has been demonstrated that an increase in carbon content leads to an increase in stress during deformation [10,14]. Given the comparable stress levels observed in the ferrite phases, it is evident that the martensite phase exerts a pronounced influence on the tensile strength and other mechanical properties of DP600 and DP1000 steels.

**Figure 5:**
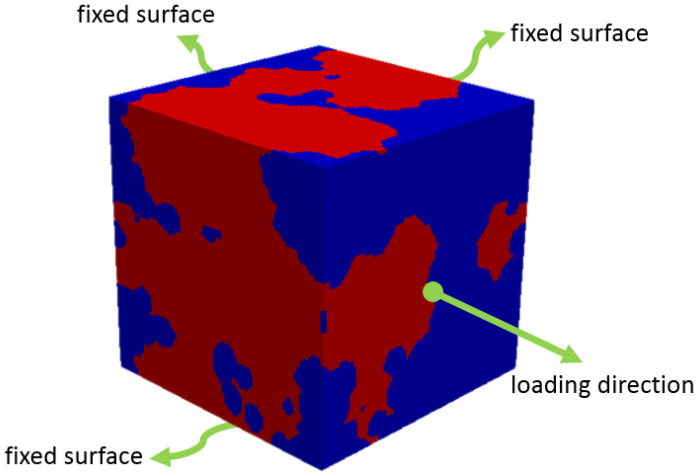
Schematic image of loading and boundary conditions on the 3D RVEs.

The symmetry axes, boundary conditions, and loading direction were identified, and the requisite deformation amount was selected. Figure 5 illustrates the axes of symmetry and the loading direction as applied to the 3D RVE model. The finite element analyses were conducted on a Dell Precision™ T7500 workstation (2 processors, 8 cores and 192 GB RAM).

## 4. Results and Discussion

**Figure 6:**
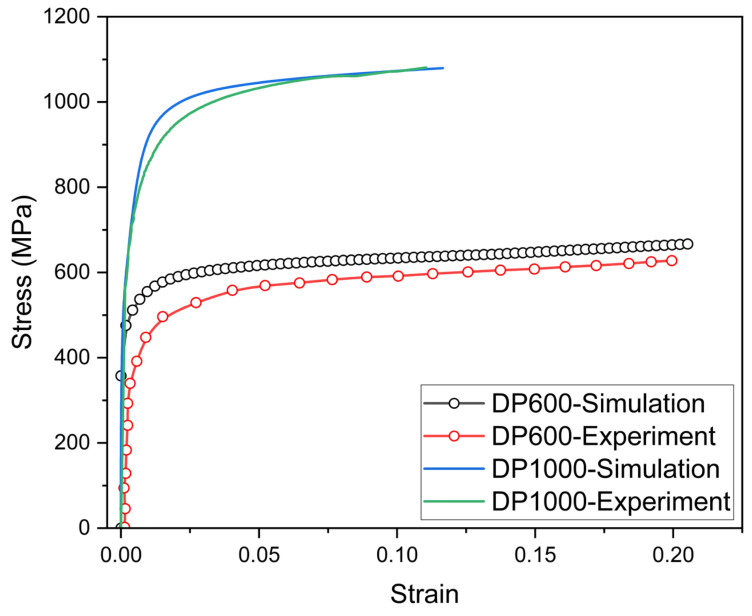
Comparison of stress–strain curve for DP600 and DP1000 dual-phase steel.

**Figure 7:**
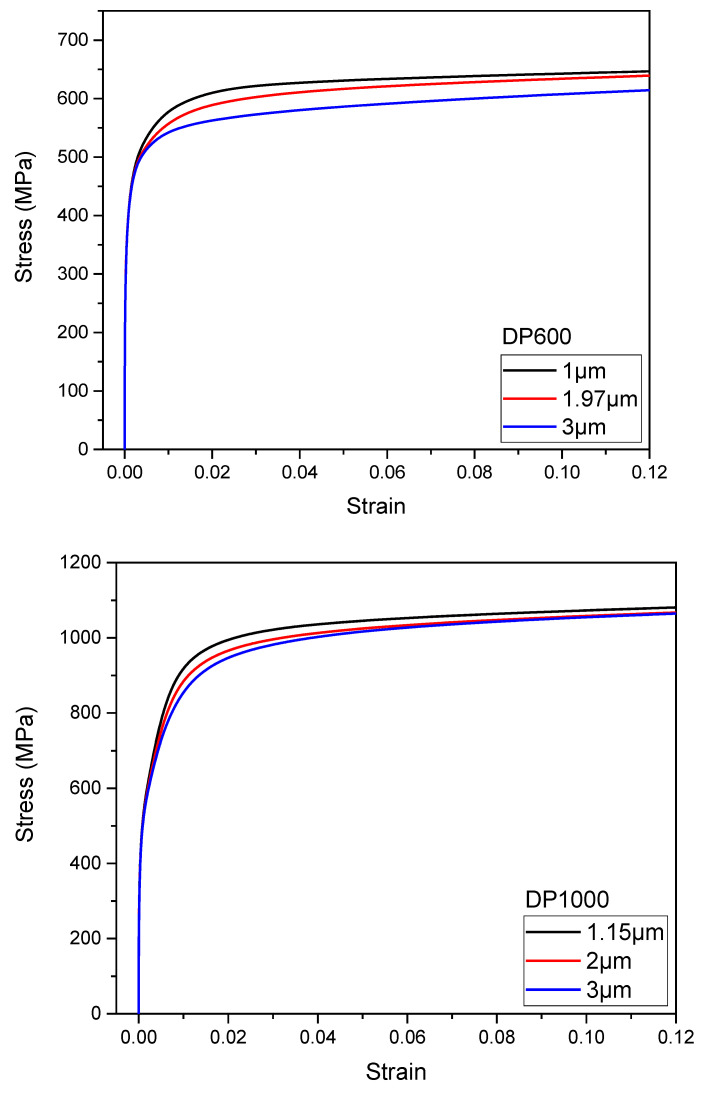
Effects of martensite particle size on tensile curves for DP600 and DP1000 dual-phase steel.

The tensile curves obtained as a result of the analyses performed using the representative volume element method for DP600 and DP1000 dual-phase steel were compared with those obtained experimentally. As illustrated in Figure 6, the representative volume element (RVE) method for DP600 and DP1000 dual-phase steel produces results that are in close agreement with the experimental data. However, the analysis results for the DP1000 dual-phase steel showed closer agreement with the experimental results. Since DP1000 dual-phase steel has a more refined martensite particle in its microstructure and a greater number of elements were used in the analytical procedures, it was postulated that the accuracy of the calculations would be improved. It was therefore surmised that the calculations would be more accurate. The effect of martensite particle size on the tensile curves at the same strain level for both steel types is shown in Figure 7. The refinement of the microstructure resulted in increased tensile strength. It is established that the refinement of the microstructure results in improved tensile strength [33]. It is evident that the presence of coarser particles results in a reduction in tensile strength. 

**Figure 8:**
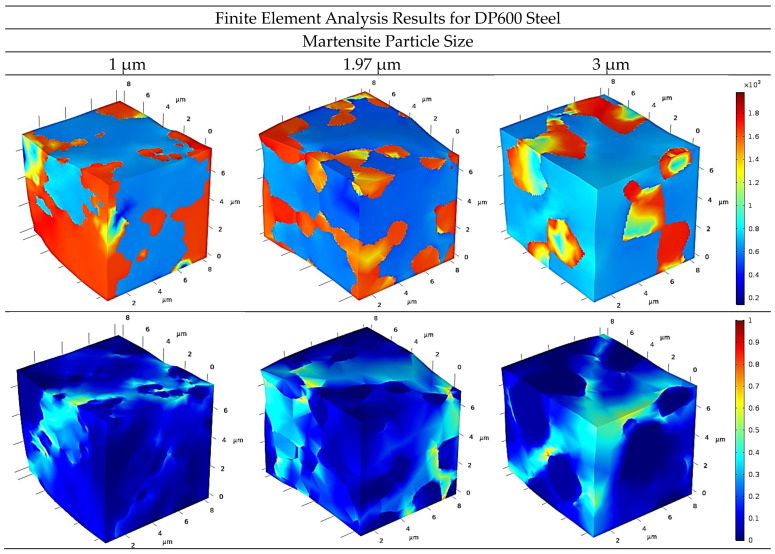
Stress–strain distribution for DP600 dual-phase steel.

**Figure 9:**
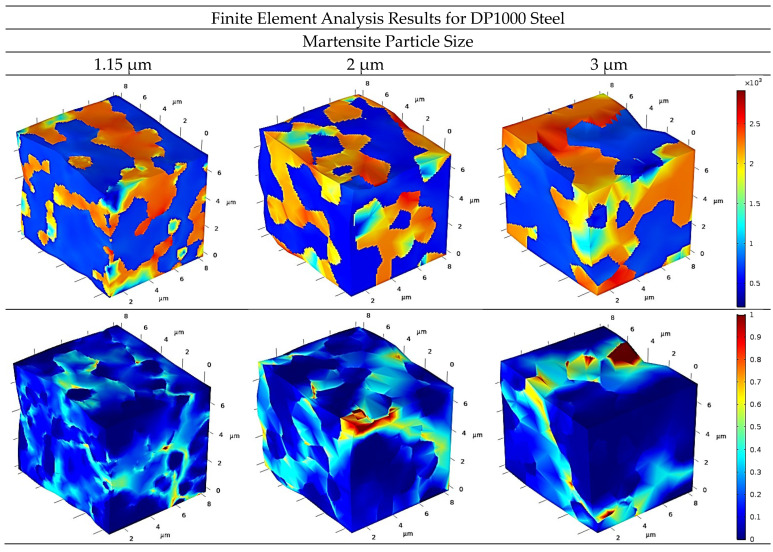
Stress–strain distribution for DP1000 dual-phase steel.

The stress deformation distributions are illustrated in Figure 8 and Figure 9 for DP600 and DP1000 dual-phase steels, respectively. The figures depict the distributions for different martensite particle sizes as three-dimensional representations. In the case of stress distributions, the yellow and red shades indicate stresses within the martensite phase, while the blue shades represent stresses within the ferrite phases. In the deformation distributions, the lightening of the blue tones serves to indicate the formation of deformation.

As illustrated in Figure 8, the intensity of the red tones diminished during the martensite phase, which can be attributed to the expansion in particle size. This can be interpreted as a reduction in stress within the martensite phase, in correlation with an increase in particle size. In contrast, the deformation distributions demonstrated that the formation of deformation increased in the ferrite phase with an increase in martensite particle size. It is assumed that this phenomenon is a consequence of the martensite phase being characterized by a coarse particle size that is unable to withstand sufficient stress. Therefore, the stress is transferred to the ferrite phase, resulting in deformation due to the phase’s insufficient strength.

Figure 9 illustrates a notable distinction between the DP600 dual-phase steel microstructure and the subject under examination. The latter displays a markedly finer and more homogeneous distribution, as evidenced by the results. The stress distributions exhibit similarities to those observed in DP600 dual-phase steel. A reduction in stress was achieved by increasing the martensite particle size, while an increase in deformation was noted with an increase in particle size. The deformation is observed in a very narrow region at a martensite particle size of 1.15 µm. It is thought that this finding indicates the presence of a finer particle size capable of storing a greater amount of stress.

**Figure 10:**
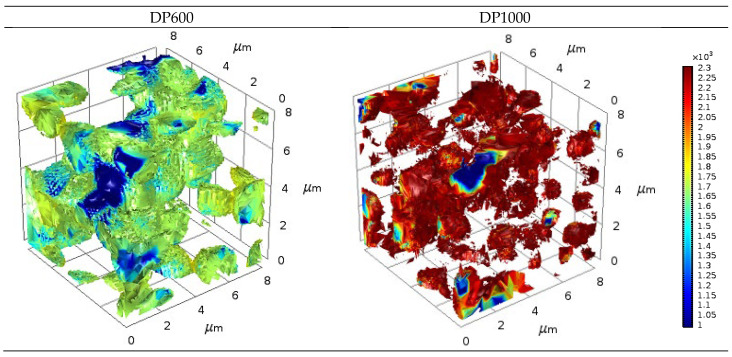
Comparison of martensite particle stress at the same loading.

Figure 10 illustrates the stress distributions on the martensite phases of the DP600 and DP1000 microstructures under the same loading conditions without using a deformation scale. The strength of the DP1000 dual-phase steel is contingent upon the quantity of carbon present, exhibiting a higher level of strength than that of the DP600 dual-phase steel. Accordingly, the stresses that occur in the martensite phase are subject to variation under the same loading conditions. The results show that the color distribution in the DP1000 dual-phase steel microstructure is predominantly reds. This indicates that the stress distribution on the martensite phase occurs at values of approximately 2300 MPa. In the DP600 dual-phase steel, the predominant colors are green tones. This indicates that the stresses experienced by the martensite phase occur at values of approximately 1700 MPa. The results of the stress distributions were found to be highly consistent with the phase tensile curve obtained, which was dependent on the chemical composition.

## 5. Conclusions

The findings of this study are summarized below.

- The mechanical behavior and, consequently, the performance of dual-phase steel are significantly influenced by the size of martensite particles.
- It was observed that the flow curves obtained from the simulation results exhibited a high degree of compatibility with the experimental results. The behavior of stress and strain in engineering applications of dual-phase steels can be predicted by means of three-dimensional representative volume elements.
- As the particle size decreased, a concomitant increase in tensile strength was observed, accompanied by a higher level of stress accumulation in the fine martensite particles. The size of martensite particles affects tensile strength since smaller particles increase the phase boundary area, which inhibits dislocation movement and thereby increases the strength of the material.
- DP1000 dual-phase steel, which exhibits a high martensite volume fraction and carbon content, demonstrated higher stress levels with less deformation than DP600 dual-phase steel.
- The mechanical interaction between the ferrite and martensite phases results in the transfer of stress across interphase boundaries, which may lead to the formation of local stress concentrations.

## Figures and Tables

**Table 1:** The chemical composition of DP600 and DP1000 dual-phase steel (wt%).

Steel	C	Mn	Cr	Si	P	S	Ni	Cu	Al
DP600	0.07	1.58	0.48	0.10	<0.02	<0.01	-	-	0.03
DP1000	0.14	1.4	<0.02	0.53	<0.01	<0.01	0.03	0.02	0.03

**Table 2:** Microstructure properties of DP600 and DP1000 dual-phase steel.

Steel	Martensite Volume Fraction	Ferrite Volume Fraction	Martensite Particle Size
DP600	29%	71%	1.97 µm
DP1000	47%	53%	1.15 µm

## Data Availability

The raw data supporting the conclusions of this article will be made available by the authors on request.

## References

[1] Schmitt J.H., Iung T. (2018). New developments of advanced high-strength steels for automotive applications. Comptes Rendus Phys..

[2] Matlock D.K., Speer J.G., Matlock D.K., Speer J.G. (2010). Processing Opportunities for New Advanced High-Strength Sheet Steels. Mater. Manuf. Process..

[3] Badkoobeh F., Nouri A., Hassannejad H. (2020). The bake hardening mechanism of dual-phase silicon steels under high pre-strain. Mater. Sci. Eng. A.

[4] Kingklang S., Julsri W., Chiyatan T., Uthaisangsuk V. (2019). A Comparative Study of Forming and Crash Behavior of High Strength Steels. Mater. Perform. Charact..

[5] Tasan C.C., Diehl M., Yan D., Bechtold M., Roters F., Schemmann L., Zheng C., Peranio N., Ponge D., Koyama M. (2015). An Overview of Dual-Phase Steels: Advances in Microstructure-Oriented Processing and Micromechanically Guided Design. Annu. Rev. Mater. Res..

[6] Çavuşoğlu O., Toros S., Gürün H. (2019). The effects of temperature and strain rate on yielding and springback behaviour of DP1000 dual phase steel. Mater. Res. Exp..

[7] Fu H., Yuan S., Sun W., Wan J., Chan K.C., Zhu J., Yang X. (2021). A novel atomic movement mechanism of intersection-induced bct-α → bcc-α′ martensitic phase transformation. Scr. Mater..

[8] Ódor É., Jóni B., Ribárik G., Chinh N.Q., Ungár T., Szabó P.J. (2020). Deformation Induced Soft and Hard Lath Packets Enhance Ductility in Martensitic Steels. Crystals.

[9] Shaning J., Zhang S., Lin J., Zhu X., Li S., Sun Y., Xia Y., Liu W., Wang C. (2023). Study on the Microstructure and Mechanical Properties of Martensitic Wear-Resistant Steel. Crystals.

[10] Çavuşoğlu O., Toros S., Gürün H. (2019). Microstructure based modelling of stress–strain relationship on dual phase steels. Ironmak. Steelmak..

[11] Paul S.K. (2013). Real microstructure based micromechanical model to simulate microstructural level deformation behavior and failure initiation in DP 590 steel. Mater. Des..

[12] Arezodar A.F., Nikbakht A. (2019). Micromechanical Modeling and Investigating the Effect of Particle Size and the Interface of Phases on the Mechanical Behavior of Dual-Phase Steels. J. Mater. Eng. Perform..

[13] Ahmad E., Manzoor T., Ziai M.M.A., Hussain N. (2012). Effect of martensite morphology on tensile deformation of dual-phase steel. J. Mater. Eng. Perform..

[14] Anbarlooie B., Hosseini-Toudeshky H., Hosseini M., Kadkhodapour J. (2019). Experimental and 3D Micromechanical Analysis of Stress–Strain Behavior and Damage Initiation in Dual-Phase Steels. J. Mater. Eng. Perform..

[15] Eghtesad A., Knezevic M. (2020). High-performance full-field crystal plasticity with dislocation-based hardening and slip system back-stress laws: Application to modeling deformation of dual-phase steels. J. Mech. Phys. Solids.

[16] Hosseini-Toudeshky H., Anbarlooie B., Kadkhodapour J. (2015). Micromechanics stress-strain behavior prediction of dual phase steel considering plasticity and grain boundaries debonding. Mater. Des..

[17] Mangas Á., Eguía I., Ezkerra J., Mendiguren J., Arechabaleta Z. (2019). Numerical study on the effect of microstructure on the mechanical behaviour of dual-phase steels. AIP Conference Proceedings.

[18] Rana A.K., Paul S.K., Dey P.P. (2018). Effect of Martensite Volume Fraction on Strain Partitioning Behavior of Dual Phase Steel. Phys. Mesomech..

[19] Li S., Guo C., Hao L., Kang Y., An Y. (2019). Microstructure-Based Modeling of Mechanical Properties and Deformation Behavior of DP600 Dual Phase Steel. Steel Res. Int..

[20] Matsuno T., Yoshioka T., Watanabe I., Alves L. (2019). Three-dimensional finite element analysis of representative volume elements for characterizing the effects of martensite elongation and banding on tensile strength of ferrite-martensite dual-phase steels. Int. J. Mech. Sci..

[21] Basantia S.K., Singh V., Bhattacharya A., Khutia N., Das D. (2018). Prediction of tensile behaviour of ferrite-martensite dual phase steel using real microstructure-based RVE simulations. Mater. Today Proc..

[22] Amirmaleki M., Samei J., Green D.E., van Riemsdijk I., Stewart L. (2016). 3D micromechanical modeling of dual phase steels using the representative volume element method. Mech. Mater..

[23] Abid N.H., Al-Rub R.K.A., Palazotto A.N. (2017). Micromechanical finite element analysis of the effects of martensite particle size and ferrite grain boundaries on the overall mechanical behavior of dual phase steel. J. Eng. Mater. Technol..

[24] Davis J.R., Testing T. (2004). ASM International, Ohio, USA. https://www.asminternational.org/wp-content/uploads/files/05106G/05106G-toc.pdf.

[25] Digital Representation Environment for Analyzing Microstructure in 3D. http://dream3d.bluequartz.net/.

[26] Groeber M.A., Jackson M.A. (2014). Groeber and Jackson Integrating Materials and Manufacturing Innovation. http://www.immijournal.com/content/3/1/5.

[27] Toros S. (2016). Microstructural finite element modeling of redox behavior of Ni-YSZ based ceramic SOFC anodes. Ceram. Int..

[28] Santos R.O., Da Silveira L.B., Moreira L.P., Cardoso M.C., Da Silva F.R.F., Paula A.D.S., Albertacci D.A. (2019). Damage identification parameters of dual-phase 600–800 steels based on experimental void analysis and finite element simulations. J. Mater. Res. Technol..

[29] Fonstein N. (2017). Dual-phase steels. Automotive Steels.

[30] Rodriguez I., Gutierrez R. (2003). Unified formulation to predict the tensile curves of steels with different microstructures. Mater. Sci. Forum..

[31] Sirinakorn T., Wongwises S., Uthaisangsuk V. (2014). A study of local deformation and damage of dual phase steel. Mater. Des..

[32] Lertkiatpeeti K., Janya-Anurak C., Uthaisangsuk V. (2024). Effects of Spatial Microstructure Characteristics On Mechanical Properties of Dual Phase Steel by Inverse Analysis And Machine Learning Approach. Computational Mater. Sci..

[33] Najafi M., Mirzadeh H., Alibeyki M. (2018). Grain Refinement of Dual Phase Steel via Tempering of Cold-Rolled Martensite. Iran. J. Mater. Form..

